# Treatment of Various Ocular Manifestation of Systemic Lupus Erythematosus with Therapeutic Plasma Exchange

**DOI:** 10.3390/jcm11226632

**Published:** 2022-11-09

**Authors:** Ji-Hye Lee, Ho-Wook Jeon, Su-Jin Moon, Mee-Yon Lee

**Affiliations:** 1 Department of Ophthalmology, College of Medicine, The Catholic University of Korea, Seoul 06591, Korea; 2 Division of Rheumatology, Department of Internal Medicine, Uijeongbu St. Mary’s Hospital, College of Medicine, The Catholic University of Korea, Uijeongbu-si 11765, Korea; 3 Department of Ophthalmology, Uijeongbu St. Mary’s Hospital, College of Medicine, The Catholic University of Korea, Uijeongbu-si 11765, Korea

**Keywords:** systemic lupus erythematosus, ocular manifestation, immunosuppressant, therapeutic plasma exchange, autoimmune

## Abstract

The goal of this study is to describe a rare case of acute systemic lupus erythematosus (SLE) ocular involvement, followed by a rapid deterioration of the overall condition, and to then describe its successful treatment with therapeutic plasma exchange (TPE). In our case, a 21-year-old female, previously diagnosed with SLE, presented with a bilateral decreased vision for one week. Fundus examination and optical coherence tomography revealed subretinal fluid accumulation in both eyes and severe disc swelling with diffuse subretinal hemorrhages and perimacular whitening in the left eye. Despite systemic high-dose steroid therapy, the patient became worse, but immunosuppressive treatment was postponed due to fever and elevated serum leukocytes with the chance of systemic infection. She had undergone therapeutic plasma exchange (TPE) and was successfully treated. Preceding SLE ocular manifestation can be an indicator of the exacerbation of SLE, and TPE can be a treatment option for such progression.

## 1. Introduction

Systemic lupus erythematosus (SLE) is a chronic, autoimmune, multisystemic disease affecting mostly women in their late teens through their forties [1,2,3]. Ocular involvement in SLE has been reported in up to one-third of patients, and symptoms can range from dry eye to vision-threatening vaso-occlusive retinopathy [1,2,3,4,5]. Among these, active retinal vasculopathy is particularly important because it is an indicator of significant disease activity and a poor prognosis [1,2,3,4,5]. According to the previous reports, retinal vasculopathy has been reported to occur from between 3% and 29%, depending on disease activity among SLE patients [1,2]. Clinically, it can range from a mild form, such as microangiopathy, to severe central retinal vessel occlusion; patients could present mild ocular discomfort all the way to a sudden loss of vision. Retinal microangiopathy depicts cotton wool spots, intraretinal hemorrhages, microaneurysms, exudates, and tortuous retinal vessels, and it could be easily misdiagnosed as diabetic retinopathy or hypertensive retinopathy [1,6]. Severe vaso-occlusive retinopathy is associated with retinal capillary nonperfusion, retinal artery or vein occlusions, vitreous hemorrhage, and ocular neovascularization [1,6]. It is rare, but holds a great clinical significance, as it can lead to visual loss; Jabs et al. reported that 55% of severe vaso-occlusive patients ended up with visual acuity worse than 20/200 [7]. Currently, systemic corticosteroids and immunosuppressants are the initial treatment of choice for significant retinal disease, and antiplatelet agents might be needed for vaso-occlusive retinopathy or for patients with significant antiphospholipid antibody elevation [6]. To prevent further retinal ischemic damage, panretinal photocoagulation and intravitreal bevacizumab should be considered, and vitrectomy surgery might be a treatment option in patients with vitreous hemorrhage.

We present a case of acute ocular involvement in SLE followed by rapid deterioration of the patient’s overall condition. Although the patient was refractory to systemic steroid treatment, she showed remarkable response to plasmapheresis.

## 2. Case Report

A 21-year-old woman came to our emergency center with seizure-like movement. She had been previously diagnosed with SLE at our rheumatologic clinic and recently stopped taking hydroxychloroquine because of drug-induced hepatotoxicity; instead, she was on a combined oral steroid and cyclosporine. The patient complained of decreased vision and mild upper lid swelling in both eyes over the past five days. During ocular examination, her best corrected visual acuity was 0.9 in the right eye and 1.0 in the left eye (Snellen decimal), and mild chemosis was noted. Fundoscopy showed no definite abnormalities, but optical coherence tomography (OCT) demonstrated focal subretinal fluid accumulation on both sides. Although she had no neurologic deficits after seizure, small acute infarctions in the left occipital cortex were revealed through brain magnetic resonance imaging (MRI), and mild diffuse cerebral dysfunction was seen on electroencephalography. Moreover, disease-specific serologic markers, such as a lupus anticoagulant and an anticardiolipin antibody, were found to be positive. As both neurologic imaging and serologic markers indicated the aggravation of disease activity, the patient was admitted for close observation and high-dose steroid therapy. Her clinical and laboratory findings were highly suggestive of a vascular thrombotic event, and low-molecular-weight heparin was used.

**Figure 1:**
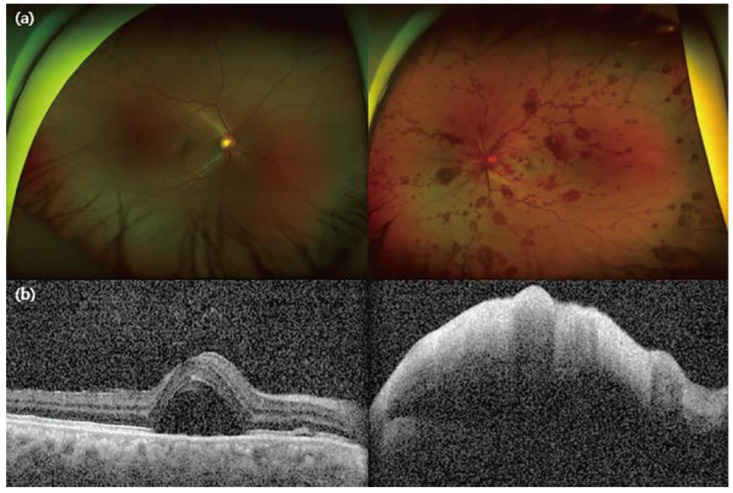
At the initial symptom presentation (**a**) funduscopy showed sudden-onset central retinal artery occlusion (CRAO) with central retinal vein occlusion (CRVO) in the left eye. (**b**) Optical coherence tomography (OCT) showed subretinal fluid (SRF) in the right eye and diffuse thickening and hyper-reflectivity of neurosensory retinal layers with marked posterior shadowing in the left eye.

**Figure 2:**
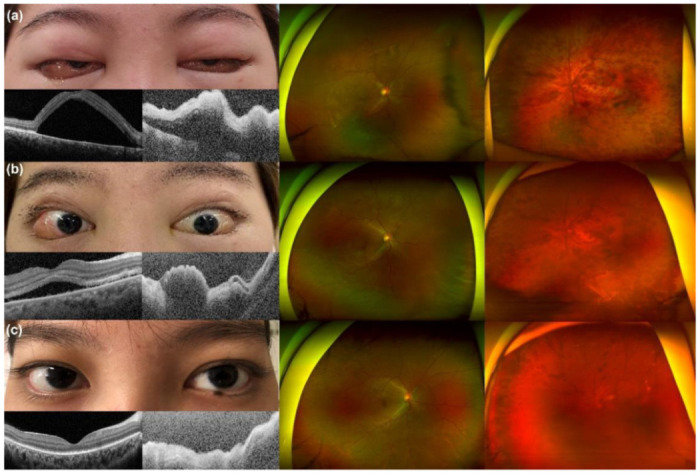
After continued intravenous corticosteroid therapy for one week, (**a**) the bilateral lid swelling and chemosis deteriorated. Moreover, the subretinal fluid (SRF) in the right eye increased. (**b**) One day after the first TPE, the bilateral lid swelling markedly improved, and SRF decreased in the right eye. (**c**) There was a complete resolution of the lid swelling, chemosis, and SRF after the sixth and last TPE.

A day later, the patient complained of sudden painless visual loss in her left eye. Visual acuity was 0.7 in the right eye with correction, and fundoscopic examination showed relatively normal retinal vasculature with serous retinal detachment at the perifoveal area. Visual acuity of the left eye was based on hand movement and non-correctable, and fundoscopy revealed a swollen optic disc with peripapillary hemorrhages, diffuse subretinal hemorrhages, a dilated and tortuous retinal vein in all four quadrants, and perimacular whitening. OCT demonstrated increased subretinal fluid collection in the right eye and diffuse thickening and hyper-reflectivity of neurosensory retinal layers with marked posterior shadowing in the left eye (Figure 1a,b). Moreover, in the left eye, fluorescein angiography revealed delayed choroidal filling and a significant artery filling defect, as well as many spots of dye leakage and pooling in the subretinal space with the right eye. Despite continued intravenous corticosteroid therapy for one week, the visual acuity reduced to hand movement on both sides, and she presented with severe bilateral eyelid swelling and bullous chemosis in both eyes (Figure 2a). Prominent limitations in extraocular movement subsequently followed. Her condition suddenly deteriorated, and she could not even tell her name properly, necessitating her transfer to the intensive care unit. Serology revealed low complement levels (C3 24.4 mg/dL, C4 2.4 mg/dL) and elevated DNA antibody, which are related to disease activity. During a follow-up MRI, no significant interval change and nothing specific to the extraocular muscles were found. Under the impression of an acute confusional state, which is a manifestation of neuropsychiatric SLE, a decision was made to perform therapeutic plasma exchange (TPE), with six treatments over two weeks. As infection could not be ruled out, combined with progressive cytopenia, other immunosuppressive treatments, such as intravenous cyclophosphamide, were postponed. Even after TPE, the visual acuity of her left eye deteriorated to light perception due to diffuse retinal artery and vein occlusion, but her right eye fully recovered; her visual acuity improved to 0.9, and the serous detachment was resolved. The patient became orientated, and the bilateral severe lid swelling and chemosis considerably decreased (Figure 2b,c).

## 3. Discussion

Ocular involvement in SLE can vary from patient to patient, as it can involve changes in the anterior segment and the posterior segment of the eye.

### 3.1. Anterior Segment

Dry eye, or keratoconjunctivitis sicca, is the most common ocular manifestation of SLE, and it is known to be associated with secondary Sjogren’s syndrome [1,2,3,6]. Jensen et al. had reported that about 60% of the patients complained of at least one symptom of dry eye [6,8]. Corneal involvement, such as corneal epitheliopathy and peripheral ulceration, can also be noted [1,2,3,6]. Episcleritis and scleritis are known to be associated with rheumatic disease, such as rheumatoid arthritis, polyarthritis nodosa, etc., but have rarely been reported as associated with SLE [1,9]. In patients with SLE, scleritis is often underdiagnosed, but it is clinically significant because it suggests that the systemic disease is in the active phase and that subsequent treatment is needed.

### 3.2. Posterior Segment

Retinal involvement is the second most common ocular manifestation following keratoconjunctivis sicca in SLE patients [2,6]. As retinal vascular micro-infarction and embolism are considered to reflect systemic vascular damage, the extent of retinal involvement is directly related to SLE disease activity index scores [10]. Additionally, even though there are other methods to evaluate the systemic vascular damage, fundus examination is the only way to directly visualize and evaluate the vascular status without damaging the tissues [10]. Common retinal findings include cotton wool spots, hard exudates, intraretinal hemorrhages, and tortuous retinal vessels [1,6]. Classified as microangiopathy, a milder form of lupus retinopathy, it is associated with a rather good visual prognosis [1,11]. However, severe vaso-occlusion, the most severe form of lupus retinopathy, depicts central retinal vessel occlusion, ocular neovascularization, tractional retinal detachment, vitreous hemorrhage, and results in vision loss [1,6]. Lupus choroidopathy is a rather less frequent form of posterior segment involvement in SLE than retinopathy. It is known to have a good visual outcome when systemic disease is controlled.

Although less frequently reported than other ocular features, eyelid involvement and orbital inflammation can be accompanied by SLE patients. Patients may complain of proptosis, chemosis, pain, and diplopia as inflammation spreads along the orbital tissues, and biopsy might be needed for an exact diagnosis [1,2].

With severe SLE ocular involvement, such as vaso-oculsive retinopathy, scleritis, and orbital inflammation, immediate systemic treatment is needed. Generally, intravenous steroid pulse therapy (methylprednisolone; 1 g/day for three to six days) is initiated and followed by oral prednisolone [2,6]. When a single steroid fails to have an effect, immunosuppressants, including cyclophosphomide, azathioprine, mycophenolate mofetil, cyclospoine, and methotrexate, are considered [6]. Sequential plasmapheresis has been found to be beneficial in people who are resistant to immunosuppressive therapy because it removes circulating immune complexes or autoantibodies that contribute to microvascular blockage [4,12,13]. According to the British Society of Rheumatology, TPE should be applied with restriction and only in rare SLE manifestations, such as thrombocytopenic purpura, catastrophic antiphospholipid syndrome, neuropsychiatric systemic lupus erythematosus, and lupus nephritis [14,15]. The current American Society for Apheresis considers TPE as a second-line treatment for SLE [14], yet ocular SLE manifestation, even its severe form, is not an indication for TPE. There have been a few reports of patients being successfully treated with plasmapheresis coupled with immunosuppressive chemotherapy, such as our patient [12,13,16]. Compared to our case, there is no other report of a single person who demonstrated such diverse ocular SLE manifestations, from severe chemosis to retinal vasculopathy, who was successfully treated with TPE and corticosteroids without other immunosuppressive agents. Although it is still debatable whether the therapeutic impact overcomes the negative effects of TPE, we demonstrated that it might be a potentially effective therapeutic option for patients who cannot undergo immunotherapy because of their medical condition.

## 4. Conclusions

This case indicates that, because SLE retinopathy reflects systemic vascular damage, a preceding SLE ocular manifestation might be an indicator of SLE exacerbation. Additionally, even though the efficacy of TPE in SLE patients is still limited, our case suggests that TPE could be a beneficial treatment option for progressed SLE.

## Data Availability

Not applicable.
